# Bridging the size gap between experiment and theory: large-scale DFT calculations on realistic sized Pd particles for acetylene hydrogenation†

**DOI:** 10.1039/d4ra03369h

**Published:** 2024-09-02

**Authors:** Apostolos Kordatos, Khaled Mohammed, Reza Vakili, Haresh Manyar, Alexandre Goguet, Emma Gibson, Marina Carravetta, Peter Wells, Chris-Kriton Skylaris

**Affiliations:** a School of Chemistry and Chemical Engineering, University of Southampton UK c.skylaris@soton.ac.uk; b School of Chemistry and Chemical Engineering, Queen's University Belfast UK; c School of Chemistry, University of Glasgow UK

## Abstract

Metal nanoparticles, often supported on metal oxide promoters, are a cornerstone of heterogeneous catalysis. Experimentally, size effects are well-established and are manifested through changes to catalyst selectivity, activity and durability. Density Functional Theory (DFT) calculations have provided an attractive way to study these effects and rationalise the change in nanoparticle properties. However such computational studies are typically limited to smaller nanoparticles (approximately up to 50 atoms) due to the large computational cost of DFT. How well can such simulations describe the electronic properties of the much larger nanoparticles that are often used in practice? In this study, we use the ONETEP code, which is able to achieve more favourable computational scaling for metallic nanoparticles, to bridge this size gap. We present DFT calculations on entire Pd and Pd carbide nanoparticles of more than 300 atoms (approximately 2.5 nm diameter), and find major differences in the electronic structure of such large nanoparticles, in comparison to the commonly investigated smaller clusters. These differences are also manifested in the calculated chemical properties such as adsorption energies for C_2_H_2_, C_2_H_4_ and C_2_H_6_ on the pristine Pd and PdC_*x*_ nanoparticles which are significantly larger (up to twice in value) for the ∼300 atoms structures. Furthermore, the adsorption of C_2_H_2_ and C_2_H_4_ on PdC_*x*_ nanoparticles becomes weaker as more C is introduced in the Pd lattice whilst the impact of C concentration is also observed in the calculated reaction energies towards the hydrogenation of C_2_H_2_, where the formation of C_2_H_6_ is hindered. Our simulations show that PdC_*x*_ nanoparticles of about 5% C per atom fraction and diameter of 2.5 nm could be potential candidate catalysts of high activity in hydrogenation reactions. The paradigm presented in this study will enable DFT to be applied on similar sized metal catalyst nanoparticles as in experimental investigations, strengthening the synergy between simulation and experiment in catalysis.

## Abstract 2

The hydrogenation of acetylene on entire large Pd/PdC_*x*_ nanoparticles has been investigated *via* large-scale DFT simulations. The impact of interstitial C has been examined whilst addressing the challenge of the simulation system size.

## Graphical abstract

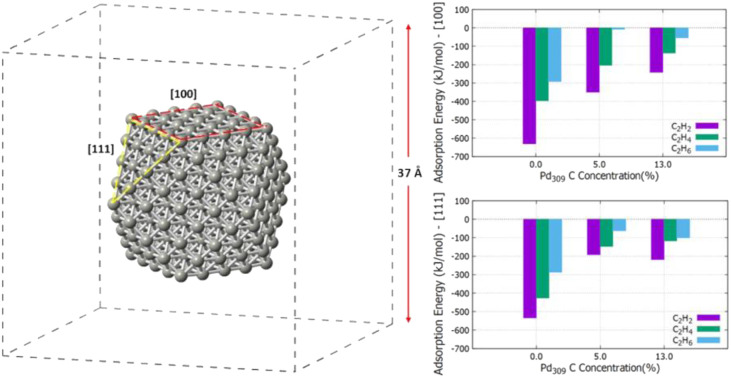

## 1. Introduction

The selective hydrogenation of acetylene has been extensively investigated as an important purification process of ethylene feedstocks in the production of polyethylene.^1–4^ During the exposure to hydrocarbons, the catalyst will adsorb acetylene, which strongly binds with Pd surface atoms. This will reduce the adsorption of ethylene whilst promoting the full hydrogenation of acetylene to ethane. Therefore, to increase selectivity towards ethylene *via* tuning the catalytic process, it is important to understand the mechanisms of ethylene and ethane formation as well as side-reactions like surface C–C oligomerization (that leads to green oil formation and deactivation of the catalyst).^5,6^

Supported Pd nanoparticles (NPs) are promising catalysts, widely used due to their high activity in a range of industrial applications^7^ such as the hydrogenation and oxidation of hydrocarbons and conversion of biomass;^8^ whilst being highly selective towards the partial hydrogenation of acetylene to ethylene. Additionally, the *in situ* formation of interstitial phases^9^ during catalysis, such as carbidic Pd,^10^ have attracted considerable interest due to their potential contribution in the increased selectivity towards the desirable products.^11^ Carbidic Pd is beneficial in blocking side-formation of phases such as hydrides,^12,13^ which otherwise would provide surface H that eventually hydrogenate ethylene to ethane. Insights on the formation mechanisms of PdC NPs are required, as well as the impact of NP size and shape with respect to the catalytic activity, aiming to tune the materials properties through controlled synthesis and achieve high stability, activity and selectivity.

The acetylene semi-hydrogenation over Pd/Al_2_O_3_ has been investigated *via* experimental and theoretical methods in the recent study of Gonçalves *et al.*^14^ where the reaction is modelled on a pyramidal Pd_30_ cluster. This study shows that full hydrogenation of C_2_H_4_ to C_2_H_6_ exhibits higher activation barriers as a first indication of Pd selectivity towards the production of ethylene. In the experimental and theoretical work of Liu *et al.*,^15^ DFT calculations show that desorption of ethylene is more favourable for the carbidic phase rather than the pristine high-coordinated Pd(111) surface. Furthermore, Vignola *et al.*^16^ investigated the C–C bond formation that leads to catalyst poisoning through the formation of oligomers. Oligomers block the active sites of the catalyst whilst consuming hydrogen that could hydrogenate acetylene to ethylene. In their study, they show that small Pd ensembles are considered as more appropriate catalysts to avoid oligomer formation, and that the particle size contribution should be further investigated as an important feature in catalytic activity. The role of subsurface C in alkyne hydrogenation has also attracted interest; in the experimental study of Teschner *et al.*,^17^ it is shown that the subsurface Pd sites filled with C or H, have a major role in the hydrogenation reactions taking place on the surface. The subsurface chemistry impact on the selective hydrogenation of ethylene has also been reported by Studt *et al.*^18^ In their theoretical study, DFT calculations have been performed showing that selectivity increases *via* weakening of the surface bond with adsorbates. In the case of Pd NPs, the size effect has been investigated by Sun *et al.*^19^ where experimental and computational work showed that C_4_ and green oil form on structures smaller than 2 nm, whilst within at a size of 2.6 nm, adsorption of ethylene becomes weaker. The C–C/C–H bonding has been investigated in the theoretical study of Zhao *et al.*,^20^ for a range of transition metal surfaces (where the most promising identified were the Pd(111) and Pt(111)), showing that the order in terms of acetylene hydrogenation activity is inverse to that of the selectivity towards ethylene. Yang *et al.*^21^ performed DFT calculations on a range of different Pd surfaces, examining the effect of subsurface C and H and showed that the close-packed Pd(111) exhibits the highest selectivity. The selective hydrogenation of acetylene in the presence of ethylene has been also investigated by Abdollahi *et al.*^22^ In their study, activation energies for the reaction process are reported for a range of Pdn (*n* = 2–15) nanoclusters. The Pd_2_ is reported to exhibit the best selectivity towards ethylene. Besides the synthesis method and characteristics such as the shape and size of the NPs, the support is also important towards activity and selectivity as reported in the experimental work by Benavidez *et al.*^23^ They showed that C supported Pd catalysts exhibit higher selectivity towards ethylene compared to oxide supported Pd catalysts. Additionally, supports such as gamma alumina may lead to green oil formation as reported by Asplund *et al.*^24^ The aforementioned works provided useful insights on the hydrogenation of acetylene on Pd catalysts as slabs and NPs, however the role of the PdC_*x*_ formation on the hydrogenation reaction in realistic systems, comparable with experimental results is still required.

In this study, we address for the first time the challenge of the simulation system size in Pd based catalysis *via* performing large-scale DFT calculations on entire large Pd/PdC_*x*_ NPs at different C concentrations. The structures used for our investigation were of more than 300 atoms and up to approximately 2.5 nm, going beyond the investigated system sizes reported so far in the literature by one order of magnitude. All geometries were fully relaxed, providing useful insights on the PdC_*x*_ formation and the effect of C concentration on the hydrogenation of C_2_H_2_. The binding modes of adsorbed C_2_H_2_, C_2_H_4_ and C_2_H_6_ on the [100]/[111] facets of pristine and carbidised structures were firstly investigated and adsorption energies for the most stable configurations were obtained. Finally, we examined the impact of interstitial C at increasing (5% and 13% per atom fraction) concentrations on the reactants, intermediates, and products of the hydrogenation of C_2_H_2_ to C_2_H_4_ and C_2_H_6_.

## 2. Methodology

The linear-scaling DFT code ONETEP^25^ was used for the modelling of the pristine Pd and PdC_*x*_ structures. For the construction of the density matrix, localized non-orthogonal Wannier functions (NGWFs) as expressed through a set of periodic sinc (p-sinc) functions^26^ were used. For these calculations, the p-sinc basis set was set to a kinetic energy cut-off of 800 eV. For the exchange and correlation interactions, the density functional of Perdew, Burke and Ernzerhof (PBE)^27^ was used together with the Grimme D2 ^28^ empirical correction for dispersion interactions. The core electrons were represented *via* norm-conserving pseudopotentials. The NGWFs and density matrix are concurrently optimized self-consistently *via* the Ensemble DFT (EDFT) method^29^ for metallic systems with a Fermi–Dirac smearing of 0.1 eV. The geometries were allowed to relax in the minimum energy configuration. For all atoms, an NGWF radius of 9.0 Bohr has been used, whilst geometry relaxations were performed at the *Γ*-point in cubic cells of 22–37 Å. The construction of Pd NPs was done using the ASE^30^ tool with the average Pd–Pd bond length of 2.74 Å. The schematic representation of the Pd and PdC_*x*_ cells is generated using the CrystalMaker^31^ software, whilst for the reaction energies between different crystallographic configurations, the following formula is used:*E*_Reaction_ = *E*_Products_ − *E*_Reactants_where *E*_Products_ corresponds to the energy of the relaxed structures of products and *E*_Reactants_ to the energy of the relaxed structures of the reactants. For the adsorption energies of C_2_H_2_, C_2_H_4_, C_2_H_6_ and intermediates on the Pd surface the following formula is used:*E*_Adsorption_ = *E*_Pd–adsorbate_ − (*E*_Pd/PdC_ + *E*_adsorbate_)where *E*_Pd–adsorbate_ corresponds to the energy of the relaxed structure of Pd NP with the adsorbate, *E*_Pd/PdC_ to the energy of the relaxed structures for the Pd/PdC_*x*_ NPs and *E*_adsorbate_ to the energy to the isolated molecule.

## 3. Results and discussion

### 3.1 Crystallography

**Fig. 1:**
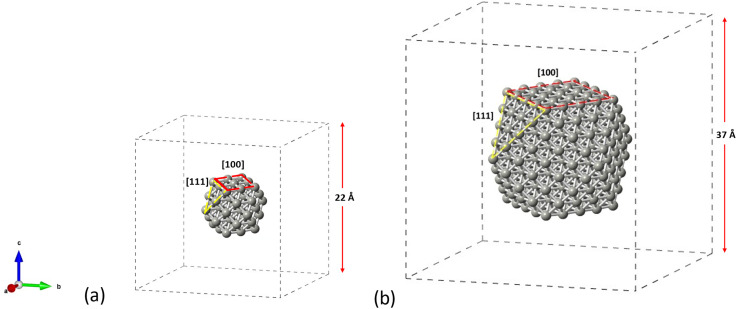
Crystallographic arrangements of the NP structures showing the cuboctahedral (a) Pd_55_ and (b) Pd_309_, as placed in the centre of their simulation boxes.

The structures considered as appropriate for this study are the cuboctahedral Pd_55_ and Pd_309_ NPs as shown in Fig. 1. We aim to account for both the [100] and [111] facets on the same particle in order to get insights on the materials performance under reaction conditions. The NP structures have been modelled in conditions of vacuum to avoid interactions with their periodic images, whilst allowing at least 5 Å of vacuum in each direction around the particle which was placed in the center of the simulation box. All structures were allowed to relax until optimized geometries were obtained. For the molecules of C_2_H_2_, C_2_H_4_ and C_2_H_6_, we performed separate geometry relaxations for the isolated structures prior to their introduction on the NP's surface.

### 3.2 Pd carbide NPs

**Fig. 2:**
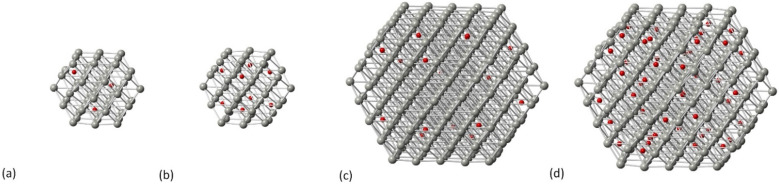
The carbidic phases of Pd NPs at (a) Pd_55_ at 5% C concentration, (b) Pd_55_ at 13% C concentration, (c) Pd_309_ at 5% C concentration and (d) Pd_309_ at 13% C concentration. Grey spheres represent Pd atoms and red spheres represent C atoms.

Carbidic Pd models were created through C incorporation corresponding to the occupation of the octahedral interstitial sites. In our previous work, we investigated the incorporation of C for different structures of Pd NPs,^32^ where we showed that there is a shape-dependent limitation of interstitial C concentration through the octahedral Pd sites; therefore, the carbidic phases considered in this work are up to a C concentration of 13% since we found that this is the maximum concentration that can be accommodated by all shapes. For the cuboctahedral structure, we have shown^32^ that the activation energy of carbidisation corresponds to 21.2 kJ mol^−1^ for the [111] facet and that, at increasing concentrations, the criterion is to allow at least one vacant site between C atoms that will preferentially occupy the subsurface area of the NP being long distance for low concentrations whilst aiming not to be in close neighboring sites at increasing amounts. In this study, two C concentrations (5% and 13%) have been considered. In Fig. 2 we show the carbidic structures for Pd_55_ and Pd_309_ whilst the average Pd–Pd bond distance for all structures is summarized in Table 1.

**Table 1:** The average Pd–Pd bond length *vs.* C concentration in cuboctahedral Pd_55_ and Pd_309_

C (%)	Pd_55_ Pd–Pd (Å)	Pd_309_ Pd–Pd (Å)
0.0	2.73	2.75
5.0	2.74	2.77
13.0	2.75	2.80

### 3.3 C_2_H_2_, C_2_H_4_ and C_2_H_6_ adsorption on Pd NPs

**Fig. 3:**
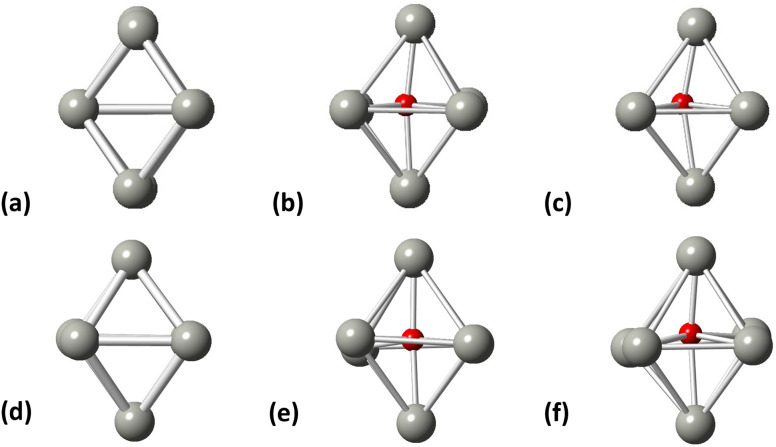
Pd octahedral interstitial sites for (a) Pd_55_ at 0% C concentration, (b) Pd_55_ at 5% C concentration, (c) Pd_55_ at 13% C concentration, (d) Pd_309_ at 0% at C concentration, (e) Pd_309_ at 5% at C concentration, and (f) Pd_309_ at 13% at C concentration. Grey spheres represent Pd atoms and red spheres represent C atoms.

**Fig. 4:**
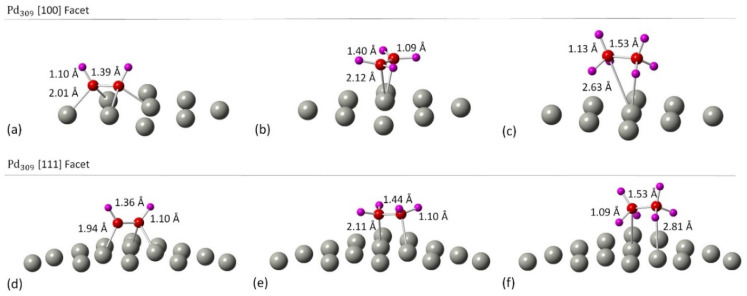
The relaxed structures of (a) C_2_H_2_ on the [100] facet of Pd_309_ NP, (b) C_2_H_4_ on the [100] facet of Pd_309_ NP, (c) C_2_H_6_ on the [100] facet of Pd_309_ NP, (d) C_2_H_2_ on the [111] facet of Pd_309_ NP, (e) C_2_H_4_ on the [111] facet of Pd_309_ NP and (f) C_2_H_6_ on the [111] facet of Pd_309_ NP. Grey spheres represent Pd atoms, red spheres represent C atoms and purple spheres represent H atoms. Geometry relaxations were performed on the entire Pd NP–ligand complexes.

**Fig. 5:**
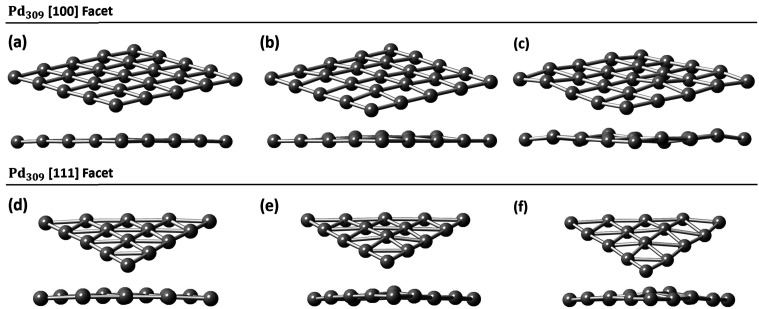
Pd_309_ surface modifications for (a) the [100] facet at 0% C, (b) the [100] facet 5% C, (c) the [100] facet 13% C, (d) the [111] facet at 0% C, (e) the [111] facet at 5% C, and (f) the [111] facet at 13% C.

**Fig. 6:**
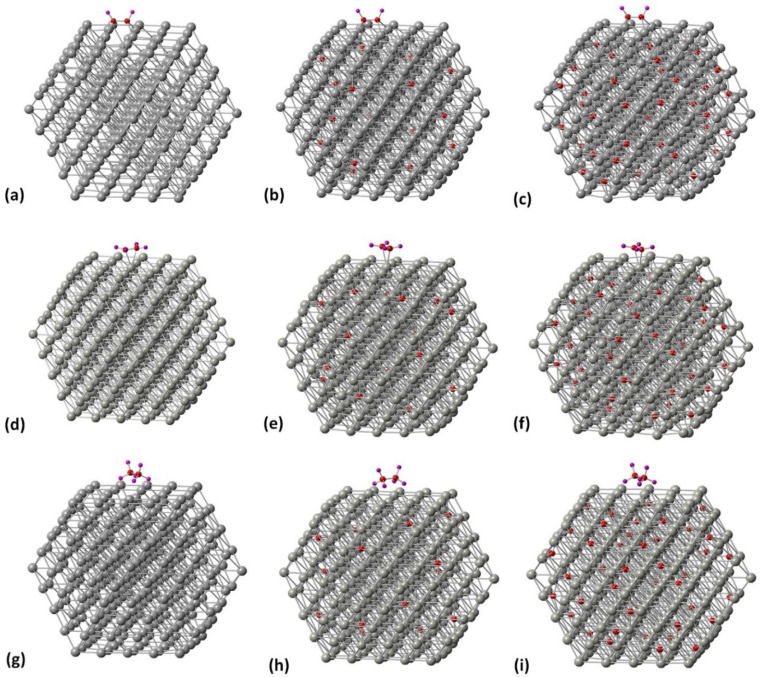
Related geometries of molecule/PdC_*x*_ NP complexes. (a)–(c) C_2_H_2_ on the [100] facet of Pd_309_ at 0, 5 and 13% C concentration, (d)–(f) C_2_H_4_ on the [100] facet of Pd_309_ at 0, 5 and 13% C concentration, and (g)–(i) C_2_H_6_ on the [100] facet of Pd_309_ at 0, 5 and 13% C concentration.

**Fig. 7:**
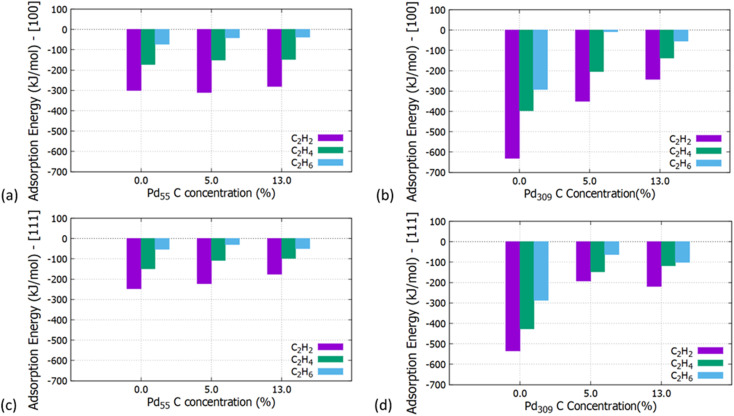
Adsorption energies of C_2_H_2_, C_2_H_4_ and C_2_H_6_ on the (a) [100] facet of Pd_55_ at C concentration = 0%, 5% and 13%, (b) [100] facet of Pd_309_ at C concentration = 0%, 5% and 13%, (c) [111] facet of Pd_55_ at C concentration = 0%, 5% and 13% and (d) [111] facet of Pd_309_ at C concentration = 0%, 5% and 13%.

Initially, we studied the preferred adsorption and orientation of the molecules on the NP surface by doing full geometry relaxations of each NP-adsorbate complex. These geometries were used subsequently as configurations of reactants and products in the partial and full hydrogenation states of C_2_H_2_ to C_2_H_4_ and C_2_H_6_. The relaxation of C in the octahedral interstitial Pd sites at 0%, 5% and 13% concentration is shown in Fig. 3. The average Pd–C bond length for Fig. 3(b) and (c) is 2.04 Å and 2.07 Å respectively whilst for 3(e) and (f) is 2.01 Å and 2.06 Å respectively. Fig. 4 shows the relaxed structures of C_2_H_2_, C_2_H_4_ and C_2_H_6_ on the [100] and [111] facets of the pristine Pd_309_ surface. The Pd_55_ relaxed structures and NP–adsorbate bond lengths for all geometries used for this study are included in the ESI, Fig. S1–S6.† Additionally, the surface modifications for the [100] and [111] facets of the Pd_309_ are also shown in Fig. 5, whilst the geometries of C_2_H_2_, C_2_H_4_ and C_2_H_6_ on the [100] facet of pristine and carbidic Pd_309_ are shown in Fig. 6. It is evident that interstitial C will change the facet morphology leading to different binding arrangements between surface Pd and hydrocarbons. We compared the adsorption energies of the C_2_H_2_ to C_2_H_4_ and C_2_H_6_ molecules at different C concentrations, NP size and facet. The adsorption energies for both [100] and [111] facets of the cuboctahedral structures are shown in Fig. 7, whilst the obtained values are presented in Tables S1 and S2 in the ESI.† Our calculations showed that C_2_H_2_ adsorbs more strongly on the 4-fold site of the [100] facet and in agreement with the work of Crespo-Quesada *et al.*^33^ We also find that C_2_H_2_ is adsorbed more strongly (−300 kJ mol^−1^ for the [100] of Pd_55_ and −631.1 kJ mol^−1^ for the [100] of Pd_309_) on the surface of the NP than C_2_H_4_ (−172.9 kJ mol^−1^ for the [100] of Pd_55_ and −396.9 kJ mol^−1^ for the [100] of Pd_309_) and C_2_H_6_ (−72.0 kJ mol^−1^ for the [100] of Pd_55_ and −291.6 kJ mol^−1^ for the [100] of Pd_309_). We observed that the adsorption energies are considerably higher for the Pd_309_ structure, showing that the particle size is expected to affect the reaction. As we introduce interstitial C in the Pd lattice, the adsorption energies are reduced drastically for Pd_309_ but very little for Pd_55_. We also observed that the carbidic Pd has significantly lower adsorption energies for C_2_H_4_ on the [100] and [111] facets as compared to the pristine structure. This shows that ethylene will desorb more easily from the surface of the PdC_*x*_ NP, hence it is a first indication of possible suppression of the full hydrogenation to ethane.

**Fig. 8:**
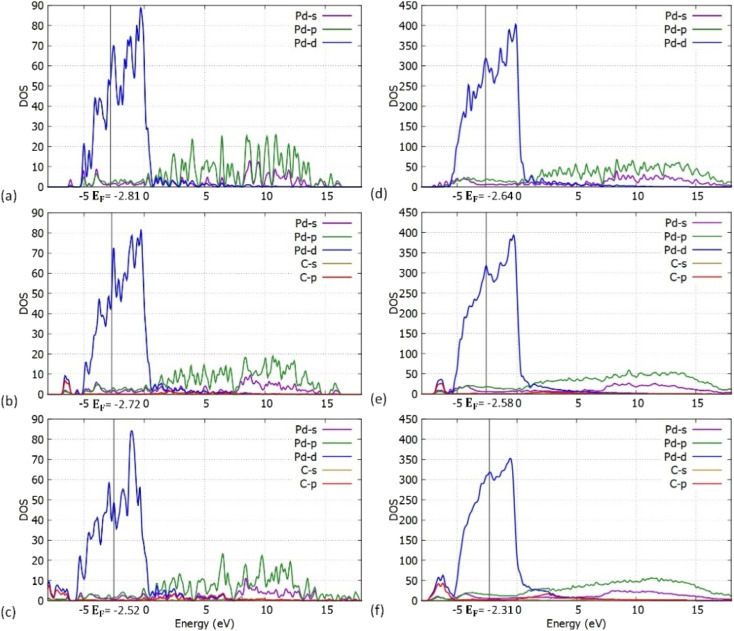
Atom and orbital resolved DOS plots for the (a) Pd_55_ at C = 0% concentration, (b) Pd_55_ at C = 5% concentration, (c) Pd_55_ at C = 13% concentration, (d) Pd_309_ at C = 0% concentration, (e) Pd_309_ at C = 5% concentration and (f) Pd_309_ at C = 13% concentration.

We have also performed density of states (DOS) investigations on the pristine and carbidic Pd_55_ and Pd_309_. As shown in Fig. 8, the metallic behaviour for both pristine structures is confirmed. Furthermore, Pd_309_ corresponds considerably more to bulk-like DOS when compared to Pd_55_. This behaviour is expected since the volume of the particle is larger, and its electronic structure is closer to bulk Pd.

To further explore the effect of C concentration on the hydrogenation of C_2_H_2_, we investigated the reaction energies of each stage of the formation of C_2_H_4_ and C_2_H_6_.

### 3.4 Hydrogenation reactions

**Fig. 9:**
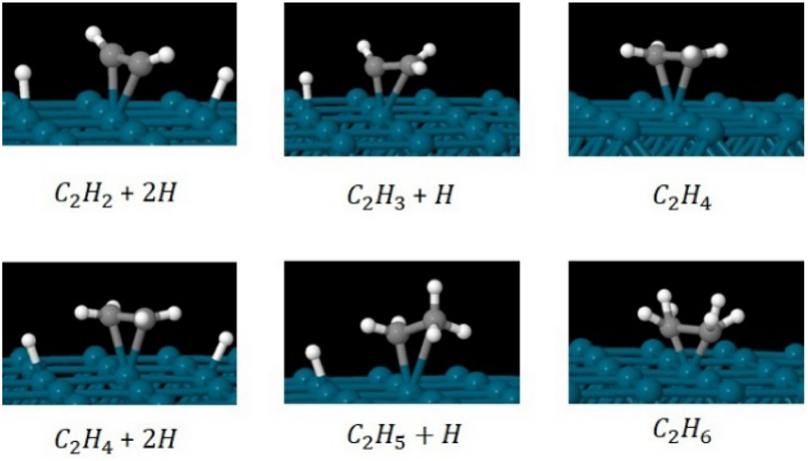
Relaxed geometries of reactants, intermediates and products for the hydrogenation of C_2_H_2_ to C_2_H_4_ (part B) and C_2_H_6_ (part D) on the [100] facet of pristine Pd_309_. Geometry relaxations were performed on the entire Pd_309_–ligand complex. Blue spheres are Pd, grey spheres are C and white spheres are H.

We investigated the partial and full hydrogenation of C_2_H_2_ on the pristine Pd_55_ and Pd_309_. Fig. 9 shows the reactants, products and intermediates on the Pd_309_ surface. The hydrogenation of acetylene on Pd NPs was investigated as a four-part process leading to partial and full hydrogenation corresponding to C_2_H_4_ and C_2_H_6_ as the two final products:(Part A) C_2_H_2_ + 2H → C_2_H_3_+H(Part B) C_2_H_3_ + H → C_2_H_4_(Part C) C_2_H_4_ + 2H → C_2_H_5_+H(Part D) C_2_H_5_ + H → C_2_H_6_

**Fig. 10:**
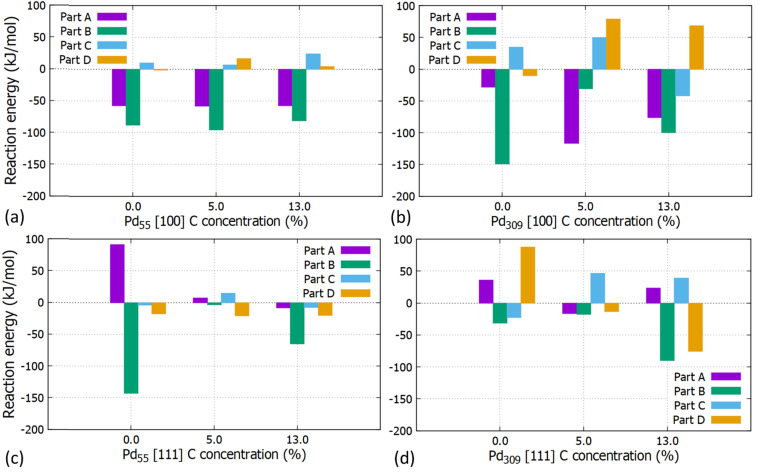
Reaction energies for the partial and full acetylene hydrogenation on the (a) [100] facet of the Pd_55_ at C (=0%, 5% and 13%), (b) [100] facet of the Pd_309_ at C (=0%, 5% and 13%), (c) [111] facet of the Pd_55_ at C (=0%, 5% and 13%) and (d) [111] facet of Pd_309_ at C (=0%, 5% and 13%).

The reaction energies for the partial and full hydrogenation of C_2_H_2_ to C_2_H_4_ and C_2_H_6_ on the [100] and [111] facets of Pd_55_ and Pd_309_ at 0, 5 and 13% of interstitial C concentration are shown in Fig. 10. Our results show that information about the catalytic behaviour of PdC_*x*_ NPs used in practical studies can be obtained by simulating entire large PdC_*x*_ NPs, as done in this work.

For Pd_55_, reaction energies for the [100] facet are exothermic for parts A (*E*_Reaction_ = −57.6 kJ mol^−1^) and B (*E*_Reaction_ = −88.1 kJ mol^−1^) whilst the introduction of interstitial C has a minor contribution. For the [111] facet, part A corresponds to endothermic reaction energies for pristine Pd and PdC_*x*_ (5%). Here, interstitial C results to lower values and the behaviour is affected by the particle's distortion at the higher C concentration of 13%.

For the pristine Pd_309_ the partial hydrogenation on the [100] facet, part B is more exothermic (*E*_Reaction_ = −149.3 kJ mol^−1^) than part A (*E*_Reaction_ = −28.6 kJ mol^−1^) for the semi-hydrogenation to C_2_H_4_. As we introduce interstitial C in the Pd lattice, C_2_H_3_ forms more easily as the reaction energy becomes more exothermic, whilst when we reach the maximum considered C concentration of 13%, reaction energies correspond to similar values. For the [111] facet, part A is also endothermic. In contrast with Pd_55_, the reaction energy turns to endothermic for 5% C concentration showing that interstitial C, larger available surface area and reduced distortion of Pd_309_ will promote the reaction. Overall, we observe that parts A and B correspond to exothermic reactions showing that the formation of intermediate C_2_H_3_ and C_2_H_4_ as the final product on the [100] facet of Pd_309_ is energetically favourable. Furthermore, the carbidic phase has a considerable impact on the reaction energies. This is due to the stronger adsorption of intermediates such as C_2_H_3_ interacting with more surface Pd atoms compared to the pristine Pd_309_. This is due to the [100] facet getting distorted as a consequence of C doping. For the full hydrogenation to C_2_H_6_ (parts C and D), our calculations showed that the reaction to C_2_H_5_ is slightly endothermic (*E*_Reaction_ = 34.82 kJ mol^−1^) and full hydrogenation to ethane corresponds to a slightly exothermic energy of reaction (*E*_Reaction_ = −10.46 kJ mol^−1^). This behaviour is in agreement with previously reported computational work^14^ where the reaction energy for the formation of C_2_H_5_ is endothermic (approximately 25 kJ mol^−1^) and the reaction energy for the formation of C_2_H_6_ is exothermic (approximately −9.6 kJ mol^−1^). The same behaviour is observed for the increasing concentration of 5% C although here the full hydrogenation to ethane also turns to endothermic.

Our calculations unambiguously show that interstitial C at a concentration of around 5% promotes the formation of ethylene, since parts C and D that would lead to ethane are endothermic. Furthermore, at this concentration, the adsorption energy of C_2_H_4_ is reduced by more than 30% compared to the pristine Pd, whilst when increasing the interstitial C concentration close to saturation (at about 13%), part C becomes exothermic again. Therefore, more work is needed towards understanding the effect of the carbidic phase to increase selectivity in Pd catalysts.

## 4. Conclusions

We performed large-scale DFT calculations on entire cuboctahedral Pd_55_ and Pd_309_ NPs with different degrees of carbidisation to gain insights on the hydrogenation of C_2_H_2_ to C_2_H_4_ and C_2_H_6_. We investigated two phases of PdC_*x*_ at concentrations of 5% and 13% (which is close to the maximum experimentally and computationally determined value) to examine how the particle size affects the adsorption and reaction energies during catalytic hydrogenation given that metal nanoparticle catalysts, often supported on metal oxide promoters, are essential to many applications in heterogeneous catalysis. Experimentally, size effects are well-established and are manifested through changes to catalyst selectivity, activity and durability and DFT calculations have provided an attractive way to study these effects and rationalise the change in nanoparticle properties. However, most DFT studies are typically limited to smaller nanoparticles (approximately up to 50 atoms) due to the large computational cost of DFT. Here we have used the ONETEP code, which is able to achieve more favourable computational scaling for metallic nanoparticles, to bridge this size gap and simulate nanoparticles of more than 300 atoms (approximately 2.5 nm diameter). In this study, we have used cuboctahedral Pd nanoparticles with 55 and 309 atoms and also their carbidised structures. We found that the adsorption energies of C_2_H_2_, C_2_H_4_ and C_2_H_6_ on the pristine Pd and PdC_*x*_ NPs are considerably larger for the Pd_309_ structure. Furthermore, the carbidic phase for the Pd_55_ has minor impact on the adsorption energies whilst for the Pd_309_, a considerable decrease is observed as more C is introduced in the Pd interstitial sites. C_2_H_2_ adsorbs more strongly on the [100] facet, forming four C–Pd bonds, rather than on the [111]. However, the adsorption of C_2_H_2_ and C_2_H_4_ becomes weaker (on Pd_309_C_*x*_) as more interstitial C is introduced with the latter being responsible for promoting desorption of ethylene and blocking full hydrogenation to ethane. Since the adsorption energies decrease for Pd_309_ with increasing C concentration, while Pd_55_ is insensitive to interstitial C, it is clear that catalytic selectivity towards partial hydrogenation can only be achieved with larger Pd NPs.

Overall, we see that the partial hydrogenation of C_2_H_2_ to C_2_H_4_ is favourable for both Pd_55_ and Pd_309_ with the exception of the [111] facet of Pd_55_ where monoatomic hydrogen relaxes towards the edges between facets making the hydrogenation reaction unfavourable. We showed that the size of the particle is expected to have a major impact on the reaction due to the available surface area where molecules adsorb as evidenced through the reaction energies. For the full hydrogenation of C_2_H_2_ we observed that the carbide phase has also an impact on the reaction towards the formation of C_2_H_5_ and C_2_H_6_, but only for Pd_309_, not Pd_55_. For a C concentration of 5%, the reaction is endothermic for both C_2_H_5_ and C_2_H_6_ for both facets preventing hydrogenation towards ethane. For a higher C concentration (13%) in Pd_309_, we found that hydrogenation to C_2_H_5_ becomes exothermic. This is an undesirable intermediate that could also lead to full hydrogenation to ethane. Therefore, the effect of the C concentration is complex, and more work is needed to tune it towards optimum selectivity and yield, but the present study provides starting points towards further optimization of the PdC_*x*_ large nanoparticle catalyst.

This is the first time that simulations on entire large (>300 atoms) metallic NPs catalysts have been performed through DFT towards understanding catalytic hydrogenation reactions. We show that there is a dramatic difference in the behaviour of large Pd NPs as compared to small Pd NPs on the adsorption energies of hydrocarbons and the reaction energies between intermediates and products. Such DFT atomistic simulations of large realistic NPs are expected to act synergistically with experimental studies to provide detailed and valuable insights in Pd-based directed catalysis.

## Data availability

The data supporting this article have been included as part of the ESI.†

## Conflicts of interest

There are no conflicts to declare.

## Supplementary Material

RA-014-D4RA03369H-s001
