# Supplementary material for: Bridging the size gap between experiment and theory: large-scale DFT calculations on realistic sized Pd particles for acetylene hydrogenation
Source: RSC Adv. 2024 Sep 2;14(38):27799–808. doi: 10.1039/d4ra03369h (PMC11367406; doi:10.1039/d4ra03369h)
Supplement: RA-014-D4RA03369H-s001 [file RA-014-D4RA03369H-s001.pdf]

Supplementary Information (SI) for RSC Advances.
This journal is © The Royal Society of Chemistry 2024

SUPPORTING INFORMATION

Table S1. Adsorption energies for C_2H2, C_2H_4, C_2H_6 vs C concentration on Pd_{55}.

**$Pd_{55}C_X$**	C_2H_2	C_2H_4	C_2H_6
**[100]**	**Adsorption kJ mol^{-1}**		
(C = 0)	-300.4	-172.9	-72.0
(C = 0.05)	-309.7	-151.0	-40.3
(C = 0.13)	-279.9	-148.0	-37.7
**[111]**	**Adsorption kJ mol^{-1}**		
(C = 0)	-247.9	-148.9	-52.1
(C = 0.05)	-222.1	-107.1	-29.3
(C = 0.13)	-176.0	-98.1	-48.9

Table S2. Adsorption energies for C_2H_2, C_2H_4, C_2H_6 vs C concentration on Pd_{309}.

**$Pd_{309}C_X$**	C_2H_2	C_2H_4	C_2H_6
**[100]**	**Adsorption kJ mol^{-1}**		
(C = 0)	-631.1	-396.9	-291.6
(C = 0.05)	-350.5	-203.9	-7.3
(C = 0.13)	-242.2	-137.6	-53.7
**[111]**	**Adsorption kJ mol^{-1}**		
(C = 0)	-534.9	-426.8	-286.8
(C = 0.05)	-192.4	-147.9	-63.0
(C = 0.13)	-218.9	-117.7	-100.2

Table S3. Reaction energies for the full C_2H_2 hydrogenation mechanism to C_2H_4 and C_2H_6 on cuboctahedral $Pd_{55}C_x$.

**[100]** – Reaction energies kJ mol^{-1}				
PdC_X	Part A	Part B	Part C	Part D
(C = 0)	-57.6	-88.1	9.9	-1.2
(C = 0.05)	-58.27	-95.7	6.5	16.7
(C = 0.13)	-57.35	-81.1	23.9	4.4
**[111]** – Reaction energies kJ mol^{-1}				
PdC_X	Part A	Part B	Part C	Part D
(C = 0)	91.5	-143.0	-4.1	-17.5
(C = 0.05)	7.1	-3.4	14.6	-20.6
(C = 0.13)	-8.4	-64.9	-7.9	-20.2

Table S4. Reaction energies for the full C_2H_2 hydrogenation mechanism to C_2H_4 and C_2H_6 on cuboctahedral $Pd_{309}C_x$.

**[100]** – Reaction energies kJ mol^{-1}				
PdC_X	Part A	Part B	Part C	Part D
(C = 0)	-28.6	-149.3	34.8	-10.5
(C = 0.05)	-116.8	-30.7	49.9	78.6
(C = 0.13)	-76.3	-99.9	-41.7	68.5
**[111]** – Reaction energies kJ mol^{-1}				
PdC_X	Part A	Part B	Part C	Part D
(C = 0)	35.9	-31.3	-22.5	87.5
(C = 0.05)	-16.5	-17.7	46.7	-13.5
(C = 0.13)	23.5	-89.7	38.8	-75.9

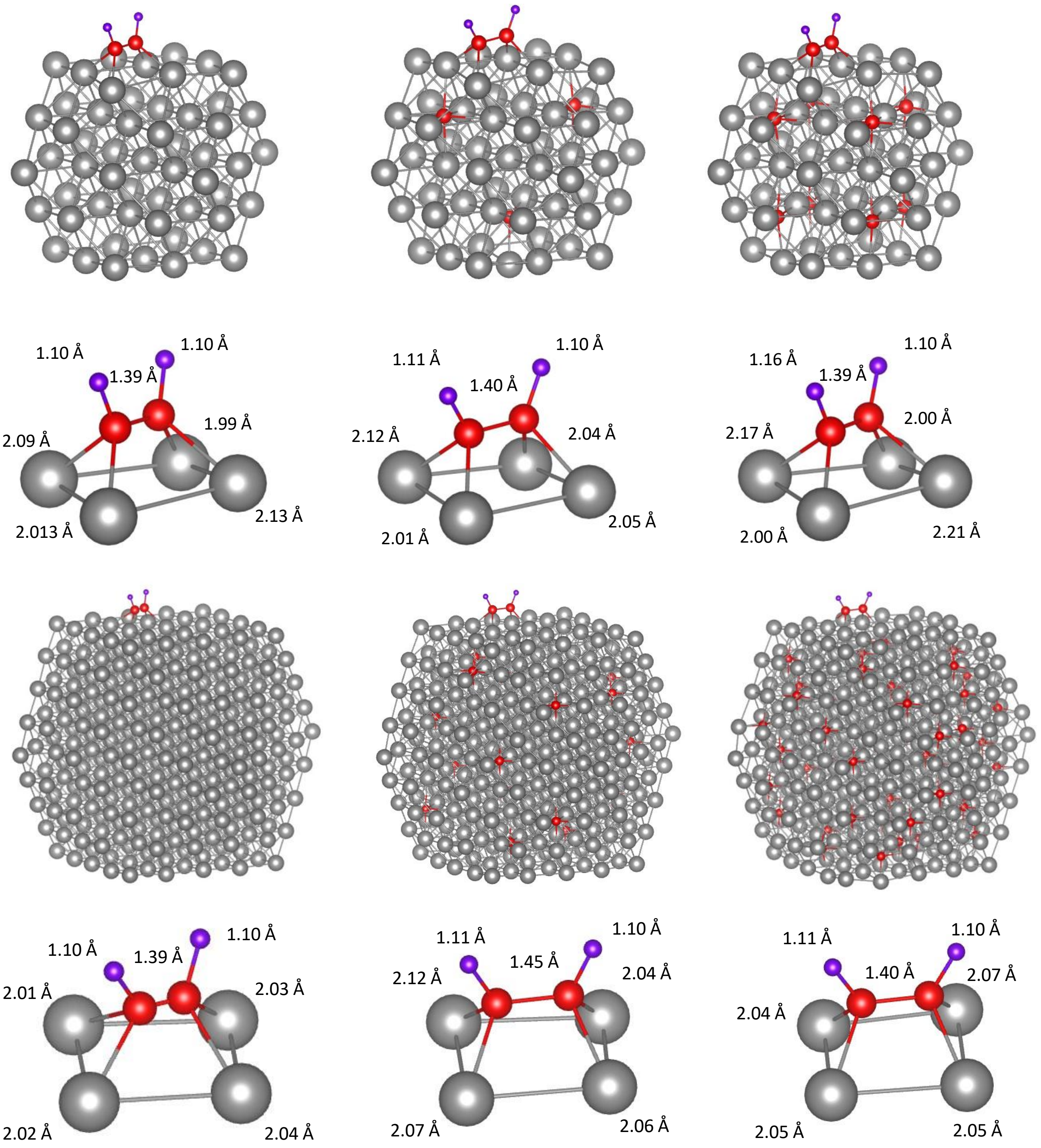

Figure S1. Acetylene bonding (Pd-C and C-H) on the [100] facet of the pristine and carbidic Pd_{55} and Pd_{309} nanoparticles.

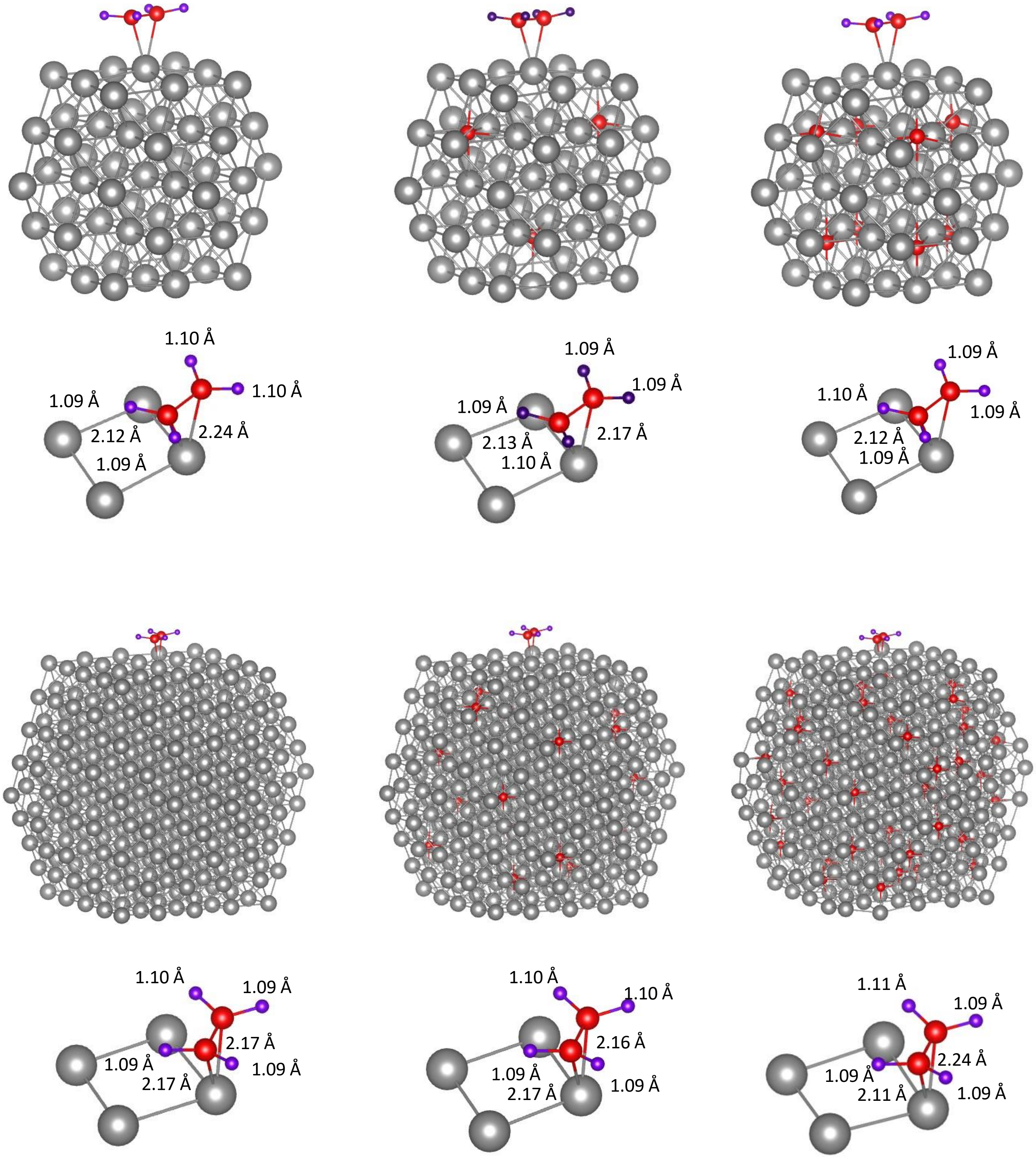

Figure S2. Ethylene bonding (Pd-C and C-H) on the [100] facet of the pristine and carbidic Pd_{55} and Pd_{309} nanoparticles.

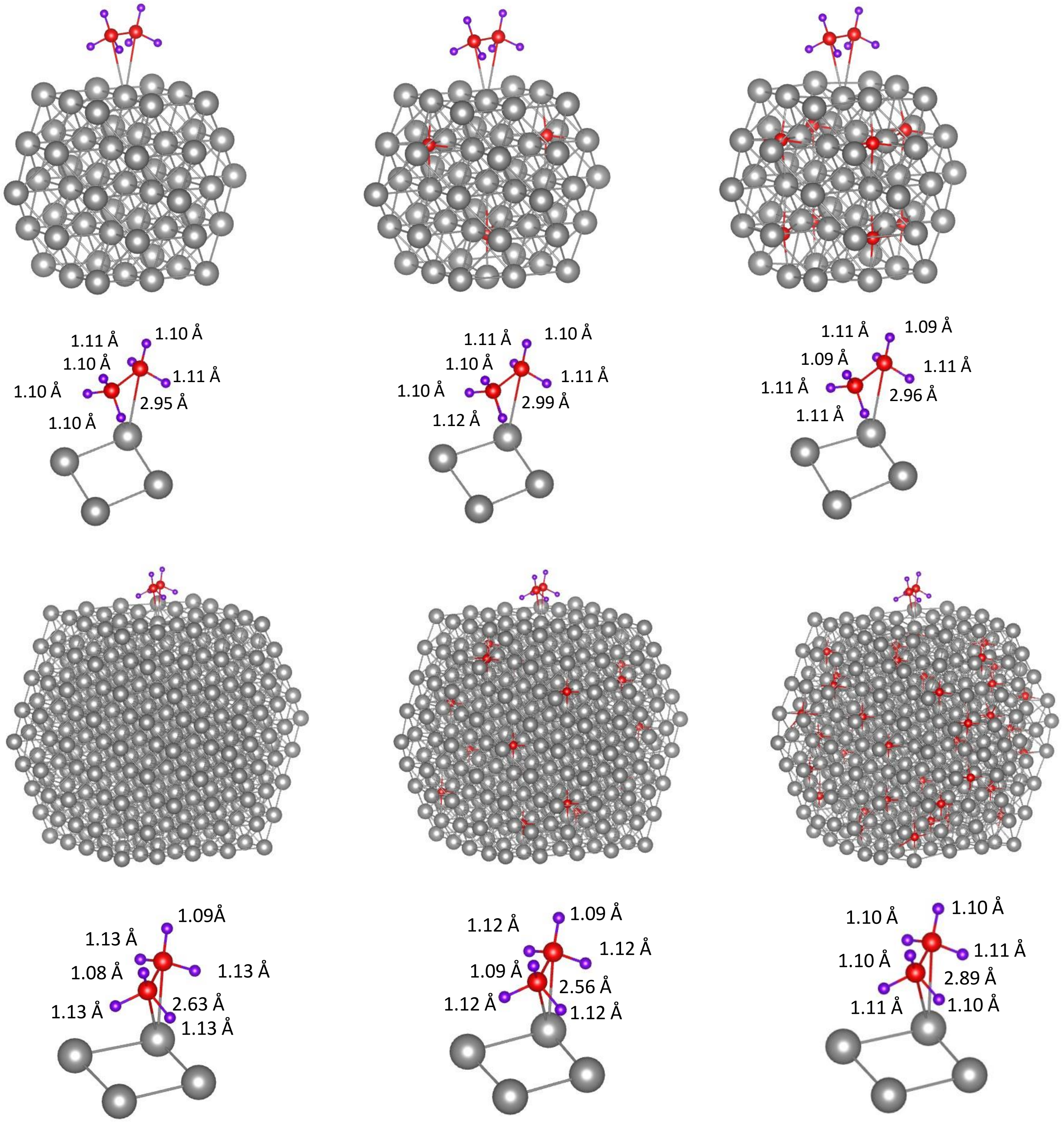

Figure S3. Ethane bonding (Pd-C and C-H) on the [100] facet of the pristine and carbidic Pd_{55} and Pd_{309} nanoparticles.

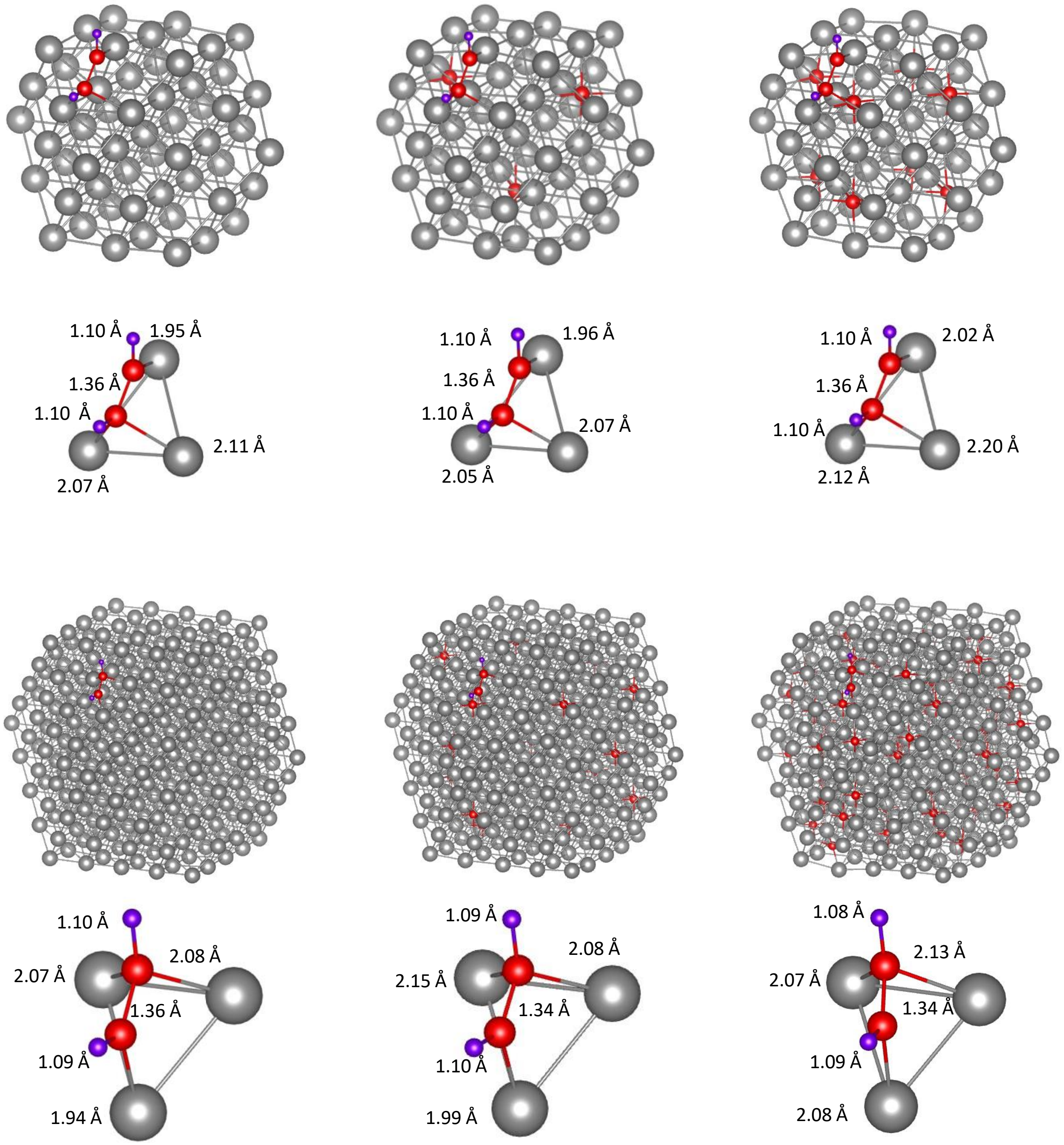

Figure S4. Acetylene bonding (Pd-C and C-H) on the [111] facet of the pristine and carbidic Pd_{55} and Pd_{309} nanoparticles.

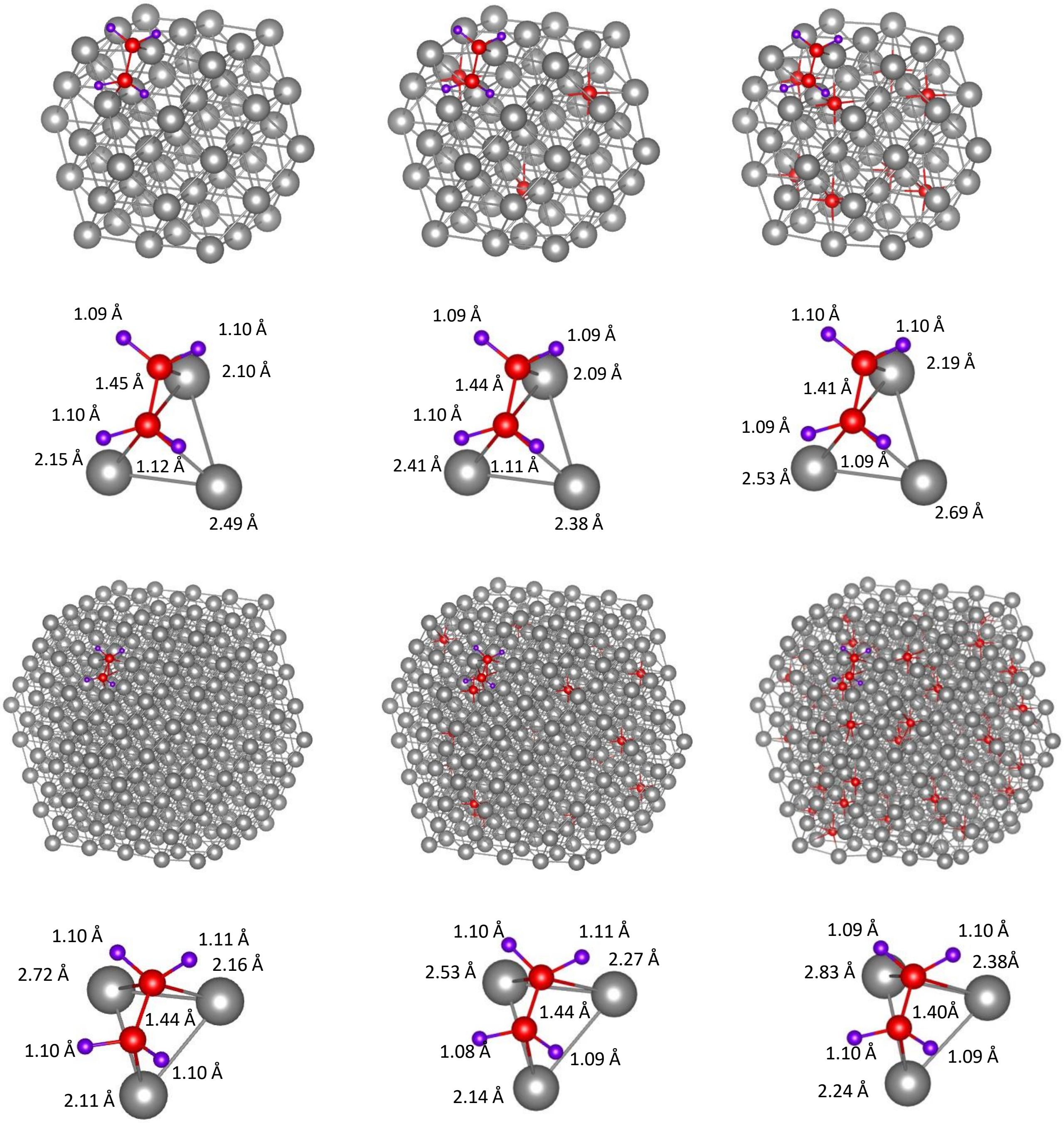

Figure S5. Ethylene bonding (Pd-C and C-H) on the [111] facet of the pristine and carbidic Pd_{55} and Pd_{309} nanoparticles.

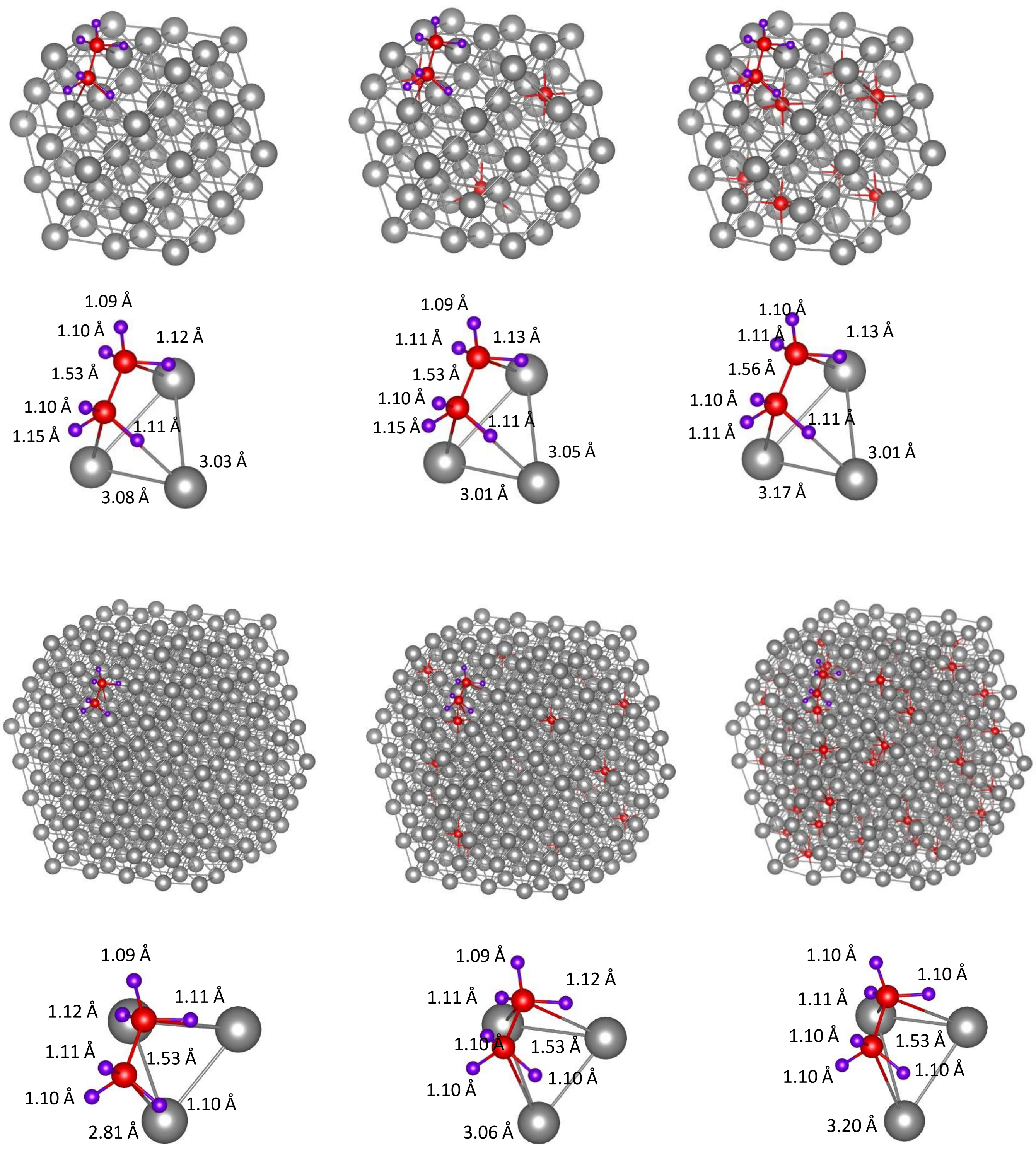

Figure S6. Ethane bonding (Pd-C and C-H) on the [111] facet of the pristine and carbidic Pd_{55} and Pd_{309} nanoparticles.
